# Unusual Type of Acinar Adenocarcinoma of the Prostate With Low PSA: A Histopathological Report of Two Cases of Pleomorphic Giant Cell Adenocarcinoma

**DOI:** 10.1155/crom/7658657

**Published:** 2025-09-09

**Authors:** George S. Stoyanov, Andrea Kirilova, Kristina Naydenova, Hristo Popov

**Affiliations:** ^1^Department of Pathology, Multiprofile Hospital for Active Treatment, Shumen, Bulgaria; ^2^Laboratory of Cytopathology, Diagnostic and Consultative Center, Shumen, Bulgaria; ^3^Department of General and Clinical Pathology, Forensic Medicine and Deontology, Faculty of Medicine, Medical University-Varna, Varna, Bulgaria; ^4^Individual Medical Diagnostic Laboratory CityLab, Varna, Bulgaria

**Keywords:** adenocarcinoma, carcinoma, giant cells, Gleason grade, Gleason score, grade group, pleomorphic and giant cell carcinoma, pleomorphic cells, prostate, prostatic carcinoma with normal or low PSA levels

## Abstract

**Introduction:** Prostate adenocarcinoma is among the leading malignant neoplastic processes in overall incidence and among the leaders of cancer-related deaths. Clinically, there is a higher risk of development in males over 50, and suspicion is increased in all cases of elevated prostate-specific antigen (PSA) levels. While the most common histologies are of conventional ductal and acinar prostatic adenocarcinoma, there are some exceedingly rare histological patterns and subtypes that vary from conventional carcinomas not only in their histological makeup but also in their presentation and aggressiveness.

**Case Presentations:** Herein, we report two cases of male patients aged 75 and 60 years who presented to our institution with the leading complaint of urinary retention and gross hematuria and concomitant hydronephrosis. In both patients, the PSA levels were within normal reference values. As such, they were scheduled for transurethral resection of the prostate, with histopathology of the resected specimens showing a pleomorphic tumor process with foci of pseudoacinar structures comprised of large atypical cells with large hyperchromic nuclei, some of which had a monstrous appearance, solid tumor cell aggregates, sheets, and single tumor cells invading the parenchyma, represented mainly by large tumor cells with macroanisokaryosis, nuclear hyperchromasia, and pronounced pleomorphism.

**Conclusion:** Pleomorphic giant cell prostatic carcinomas rarely present with elevated PSA or specific imaging findings and are often diagnosed incidentally, further contributing to their rarity.

## 1. Introduction

Prostatic adenocarcinoma is the fourth most common malignant disease overall and the eighth in cancer-related deaths worldwide [1]. In males, prostate carcinoma is a close second in overall incidence after pulmonary carcinomas and the fifth in cancer-related deaths [1].

Malignancies of the prostate generally tend to develop in males over the age of 50, and incidence is significantly higher in regions with either a very high or high human development index, with Europe and North America being the leading geographical regions, accounting for nearly half of cases [2]. These could be related to lifestyle and the availability of screening and diagnostic modalities in highly developed regions. In contrast, in underdeveloped regions, the true incidence of prostatic adenocarcinoma could remain underreported due to a lack of specific healthcare access. Furthermore, a large number of prostatic adenocarcinomas remain silent and do not manifest clinically; hence, the incidence of prostatic malignancies in autopsy cohorts is significantly higher than the reported rates in the general population, especially for males younger than 50 [3, 4].

Screening for prostatic malignancies includes predominantly prostate-specific antigen (PSA) [5]. Although PSA is organ-specific, being produced by the epithelial cells of the prostate and seminal vesicles, it is not tumor-specific, and elevated values can be seen in hyperplasia, trauma, and inflammation, although significantly less so than in malignant conditions. Furthermore, a not-so-insignificant number of malignancies can also present with normal PSA levels; these include the broad group of nonepithelial (stromal and mesenchymal) and neuroendocrine malignancies, adenoid cystic carcinoma, squamous cell carcinoma, adenosquamous carcinoma, and around 10% of higher grade conventional adenocarcinomas [6, 7]. Due to these, detailed clinical history and investigations, as well as imaging modalities such as ultrasound and magnetic resonance imaging, also play an important role in the diagnosis of prostate adenocarcinoma [8–10].

Definitive diagnosis is carried out through histopathology to establish the malignancy type—mesenchymal (stromal), epithelial (neuroendocrine, acinar, ductal, or other), or metastatic/infiltrative [11, 12]. Crucial for the following treatment for biopsy-proven epithelial malignancies is the Gleason score, Gleason grade, and grade group [13].

The two main types of prostatic adenocarcinoma are ductal and acinar, with acinar adenocarcinomas predominating, having an overall incidence of around 95% and having several exotic histological patterns and subtypes.

## 2. Case 1

A 75-year-old male presented to our institution with dysuria and polyuria. Previous medical history was significant for well-controlled Type 2 diabetes for the past 16 calendar years and hypertension for the previous 23 calendar years with suboptimal control and congestive heart failure for the previous 3 calendar years. The patient had been diagnosed and treated in an outpatient setting for benign prostate hyperplasia for the previous 7 calendar years, with regular monitoring of PSA levels, with values ranging from 0.8 to 1.003 ng/mL. Two years prior, the patient presented to our institution with gross hematuria, and a urinary bladder endoscopy showed a small, finely branching lesion on the posterior bladder wall, with histopathology showing a low-grade urothelial carcinoma with submucosal invasion (pT1). The patient was referred to the oncological committee, and intravesical therapy was initiated, with the patient remaining stable without disease recurrence and progression. Outpatient urology had suggested a prostate biopsy; however, due to the normal range of PSA levels, the patient had declined multiple times.

On the current presentation, due to significant urinary retention and imaging modalities showing Stage 2 hydronephrosis, with bloodwork also showing elevation in serum creatinine of 411 *μ*mol/L and serum urea of 24.6 *μ*mol/L, the patient agreed to transurethral resection of the prostate (TURP). Imaging before TURP showed a diffusely enlarged and irregularly contoured prostate without focal lesions.

TURP under general anesthesia went uncomplicated, and the postoperative period was uneventful. Serum creatinine and urea gradually improved. Sectioning of the TURP specimens showed multiple fragmented pieces, grayish-white and firm.

### 2.1. Histological Findings

**Figure 1:**
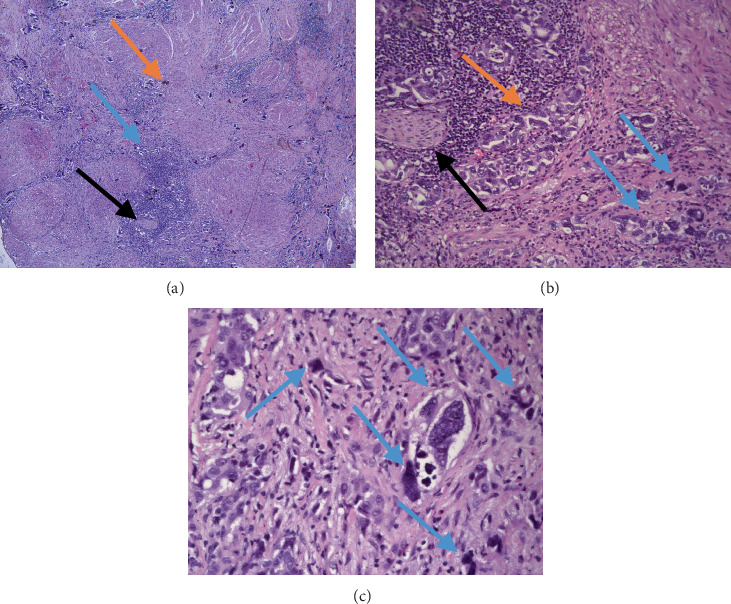
Histopathology of the tumor. (a) A heterogeneous tumor formation with giant and monstrous cells (orange arrow) intersecting the prostate parenchyma (blue arrow) with perineural invasion (black arrow), H&E stain, original magnification 50×. (b) Higher power magnification of the same field with perineural invasion (black arrow), tumor component with primitive glandular formation (orange arrow) and giant and monstrous cells (blue arrows), H&E stain, original magnification 200×. (c) Higher power view with giant and monstrous cells (blue arrows), H&E stain, original magnification 400×. H&E, hematoxylin and eosin.

Histopathology of the fragments showed fragments of prostatic parenchyma, some with pronounced necrobiotic changes involved by a pleomorphic tumor process with foci of pseudoacinar structures lined by markedly atypical cells with large hyperchromic nuclei, some of which had a monstrous appearance and comedo-type necrosis in the lumens, solid tumor cell aggregates, sheets, and single tumor cells invading the parenchyma, represented mainly by large tumor cells with macroanisokaryosis, nuclear hyperchromasia, and pronounced pleomorphism with foci of perineural invasion (Figure 1).

Based on the histopathological findings, the diagnosis of pleomorphic giant cell carcinoma of the prostate was established with a Gleason score of 5 + 5 = 10, Grade Group 5, with perineural invasion.

As such, the patient was referred to the oncological committee for further evaluation and treatment.

## 3. Case 2

A 60-year-old male presented to our institution with a 2-week history of gross hematuria and new onset intermittent pelvic pain. Previous medical history included well-controlled hypertension for an undisclosed period, prostate hyperplasia diagnosed 2 years prior, treated with alpha blockers. No documentation on PSA levels was presented upon admission; however, the patient stated that they had never been elevated. On presentation, deep vein thrombosis was noted, and laboratory tests were uneventful apart from elevated serum creatinine—131 *μ*mol/L. Imaging revealed an enlarged homogeneous prostate measuring 50 × 52 *mm* with a lesion protruding to the urinary bladder, second-stage hydronephrosis, and an enlarged 14 mm lymph node in the left external iliac group. Due to the gross hematuria and data of urinary obstruction with hydronephrosis, an endoscopy with TURP was scheduled, which was performed under general anesthesia and went uncomplicated. The postoperative period was uneventful.

### 3.1. Histological Findings

**Figure 2:**
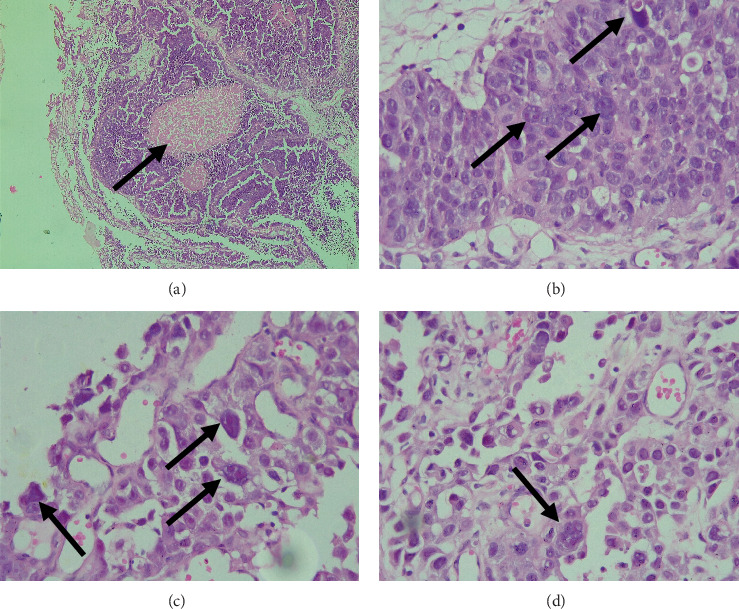
Histopathology of the tumor. (a) A heterogeneous tumor formation with central comedo-type necrosis (arrow), H&E stain, original magnification 40×. (b, c) Higher power magnification with pleomorphic nuclei and pronounced nuclear hyperchromasia (arrows), H&E stain, original magnifications 200×. (d) Focal multinucleated cells (arrow), H&E stain, original magnification 200×. H&E, hematoxylin and eosin.

Sectioning of the TURP specimens showed multiple fragmented pieces, grayish-white and firm. Histology showed multiple erythrocyte coagulums and fragments of prostate parenchyma with hyperplastic changes involved by a tumor process growing as solid nests with comedo-type necrosis in the center and solid cords comprised of stratified epithelial cells with pronounced polymorphism and nuclear hyperchromasia and individual multinucleated cells, comprising a little bit more than 5% of the total tumor parenchyma (Figure 2).

Based on the histopathology, the tumor was interpreted as pleomorphic giant cell carcinoma of the prostate with a Gleason score of 5 + 5 = 10, Grade Group 5. The patient was referred for outpatient vascular surgery for his deep vein thrombosis and to the oncological committee for further evaluation and treatment.

## 4. Discussion

Unusual histological patterns in the histopathological diagnosis of prostate adenocarcinoma, predominantly the acinar type, refer to growth patterns or cellular types that can easily be misidentified as benign processes such as atrophy, hyperplasia, or inflammation [14]. These include atrophic adenocarcinoma, which can easily be misidentified as glandular atrophy, and immunohistochemistry and molecular tests do not always indicate malignancy. This pattern is characterized by well-formed glands covered with atypical cells with loss of cytoplasmic volume intersecting the stroma of the gland; as such, this pattern is most often assigned a Gleason grade of 3 [14]. The pseudohyperplastic type is characterized by crowded glands with papillary invaginations, covered by cylindrical epithelia with abundant cytoplasm. It predominantly features nuclear atypia and invasive growth [14]. The microcystic pattern is similar to that of atrophic with cystically dilated invasive glands, covered by atypical cells with cytoplasmic reduction and nuclear features of atypia [15]. The foamy gland pattern is characterized by a xanthomatous transformation of the epithelia covering the gland with pyknotic nuclei and limited features of nuclear atypia; invasive growth is again present [14]. The mucinous pattern is characterized by at least 25% of the tumor showing extracellular mucin production, akin to intestinal-type mucinous malignancies. As such, the tumor can often be misidentified as a metastatic or directly invasive intestinal malignancy to the gland [14].

Prostatic adenocarcinoma subtypes, on the other hand, refer to tumors with both specific architectural and cellular features. These include signet-ring adenocarcinoma, again akin to the intestinal-type malignancy, wherein the malignant cells show nuclear margination by a cytoplasmic vacuole and architectural patterns predominantly of higher Gleason scores; to fit this subtype, the tumor must contain at least a 25% signet-ring-like component [14]. The sarcomatoid subtype exhibits a biphasic cellular population, one of which has a frank sarcoma morphology with multiple differentiation paths. It requires a broad differential diagnosis with metastatic prostatic stromal tumors and nonprostatic mesenchymal tumors that can develop throughout the body; immunohistochemistry is often of aid despite the sarcoma component not being positive for prostate differentiation markers [14]. Prostatic intraepithelial neoplasia-like carcinoma is a subtype characterized by dilated glands covered with more than two layers of atypical cells, albeit with limited atypical features. The invasive glandular growth pattern and immunohistochemistry for basal markers are also diagnostic aids in this subtype [14, 16].

The last recognized subtype is the abovementioned pleomorphic giant cell prostatic adenocarcinoma. This subtype is exceedingly rare, most often reported in case reports, as in this instance or limited and small cohorts, with one of the largest cohorts published encompassing only 30 cases [17]. Demographics of this subtype do not vary significantly; however, there is a reported incidence in patients younger than 40, although most cases are males aged 70 or older [17–20]. Diagnostic criteria for this subtype are the morphological presence of extensive nuclear atypia and pleomorphism, including bizarre and monstrous mononuclear and multinucleated cells with abundant cytoplasm and often abundant atypical mitoses. The distribution of these cells varies within the tumors, with some tumors exhibiting only a minor portion of the tumor parenchyma comprised of them (around 5%–10%) admixed with conventional high Gleason grade patterns as seen in the second case, while others, as in our first case, the predominant cellular component of the tumors is comprised of them [21].

Furthermore, extraprostatic extension and perineural invasion are common occurrences in this subtype as well as in combination with other subtypes [21, 22]. Clinical suspicion for malignancy is also low, as this subtype can present with normal or only slightly elevated PSA levels, which corresponds to low PSA positivity of the tumor on immunohistochemistry [21–23]. Patient survival is another significantly varying feature of this subtype, as it is exceedingly aggressive, with most reports depicting a significantly shorter survival compared to conventional acinar prostatic adenocarcinoma [21–23].

While conventionally PSA levels are used in an outpatient setting for patient risk stratification, as seen in our cases, they do not always correspond to the presence of malignancy. As such, early detection programs should also include imaging modalities. In cases where imaging features are suggestive, prostate biopsy should be performed, even in the context of low PSA levels, at least on a case-by-case basis. As such, inevitably, the incidence of low PSA-level adenocarcinomas of the prostate will increase even in other types than pleomorphic giant cell carcinoma, such as neuroendocrine tumors.

## 5. Conclusion

Pleomorphic giant cell carcinoma of the prostate is a rare and aggressive subtype of acinar prostatic adenocarcinoma. This entry's unique clinical course is defined by often normal or only slightly increased PSA levels, coexistence with other subtypes and patterns, as well as a high percentage of cases presenting with perineural invasion and extraprostatic extension. Although limited by the rarity of the diagnosis, patient survival is also shorter than that of conventional prostatic adenocarcinoma. Such rare entries are often missed in early detection programs when relying solely on monitoring of PSA levels.

## Data Availability

The data that support the findings of this study are available on request from the corresponding author. The data are not publicly available due to privacy or ethical restrictions.

## References

[1] Sung H., Ferlay J., Siegel R. L. (2021). Global Cancer Statistics 2020: GLOBOCAN Estimates of Incidence and Mortality Worldwide for 36 Cancers in 185 Countries. *CA: A Cancer Journal for Clinicians*.

[2] Center M. M., Jemal A., Lortet-Tieulent J. (2012). International Variation in Prostate Cancer Incidence and Mortality Rates. *European Urology*.

[3] Sakr W. A., Haas G. P., Cassin B. F., Pontes J. E., Crissman J. D. (1993). The Frequency of Carcinoma and Intraepithelial Neoplasia of the Prostate in Young Male Patients. *Journal of Urology*.

[4] Zlotta A. R., Egawa S., Pushkar D. (2013). Prevalence of Prostate Cancer on Autopsy: Cross-Sectional Study on Unscreened Caucasian and Asian Men. *Journal of the National Cancer Institute*.

[5] Catalona W. J., Smith D. S., Ratliff T. L. (1991). Measurement of Prostate-Specific Antigen in Serum as a Screening Test for Prostate Cancer. *New England Journal of Medicine*.

[6] Ahuja A., Das P., Kumar N., Saini A. K., Seth A., Ray R. (2011). Adenoid Cystic Carcinoma of the Prostate: Case Report on a Rare Entity and Review of the Literature. *Pathology, Research and Practice*.

[7] Epstein J. I., Egevad L., Humphrey P. A., Montironi R. (2014). Best Practices Recommendations in the Application of Immunohistochemistry in the Prostate. *American Journal of Surgical Pathology*.

[8] Rosoff J. S., Prasad S. M., Savage S. J. (2013). Ultrasonography in Prostate Cancer: Current Roles and Potential Applications in Radiorecurrent Disease. *World Journal of Urology*.

[9] Kasivisvanathan V., Rannikko A. S., Borghi M. (2018). MRI-Targeted or Standard Biopsy for Prostate-Cancer Diagnosis. *New England Journal of Medicine*.

[10] van der Leest M., Cornel E., Israël B. (2019). Head-to-Head Comparison of Transrectal Ultrasound-Guided Prostate Biopsy Versus Multiparametric Prostate Resonance Imaging With Subsequent Magnetic Resonance-Guided Biopsy in Biopsy-Naïve Men With Elevated Prostate-Specific Antigen: A Large Prospective Multicenter Clinical Study. *European Urology*.

[11] Magi-Galluzzi C. (2018). Prostate Cancer: Diagnostic Criteria and Role of Immunohistochemistry. *Modern Pathology*.

[12] Humphrey P. A. (2007). Diagnosis of Adenocarcinoma in Prostate Needle Biopsy Tissue. *Journal of Clinical Pathology*.

[13] Epstein J. I., Allsbrook WC Jr, Amin M. B., Egevad L. L., ISUP Grading Committee (2005). The 2005 International Society of Urological Pathology (ISUP) Consensus Conference on Gleason Grading of Prostatic Carcinoma. *American Journal of Surgical Pathology*.

[14] Li J., Wang Z. (2016). The Pathology of Unusual Subtypes of Prostate Cancer. *Chinese Journal of Cancer Research*.

[15] Yaskiv O., Cao D., Humphrey P. A. (2010). Microcystic Adenocarcinoma of the Prostate: A Variant of Pseudohyperplastic and Atrophic Patterns. *American Journal of Surgical Pathology*.

[16] Hameed O., Humphrey P. A. (2006). Stratified Epithelium in Prostatic Adenocarcinoma: A Mimic of High-Grade Prostatic Intraepithelial Neoplasia. *Modern Pathology*.

[17] Alharbi A. M., De Marzo A. M., Hicks J. L., Lotan T. L., Epstein J. I. (2018). Prostatic Adenocarcinoma With Focal Pleomorphic Giant Cell Features. *American Journal of Surgical Pathology*.

[18] Lopez-Beltran A., Eble J. N., Bostwick D. C. (2005). Pleomorphic Giant Cell Carcinoma of the Prostate. *Archives of Pathology & Laboratory Medicine*.

[19] Parwani A. V., Herawi M., Epstein J. I. (2006). Pleomorphic Giant Cell Adenocarcinoma of the Prostate: Report of 6 Cases. *American Journal of Surgical Pathology*.

[20] Larnaudie L., Compérat E., Conort P., Varinot J. (2017). HOXB13 a Useful Marker in Pleomorphic Giant Cell Adenocarcinoma of the Prostate: A Case Report and Review of the Literature. *Virchows Archiv*.

[21] Bilé-Silva A., Lopez-Beltran A., Rasteiro H. (2023). Pleomorphic Giant Cell Carcinoma of the Prostate: Clinicopathologic Analysis and Oncological Outcomes. *Virchows Archiv*.

[22] Fakhralddin S., Ali R., Abdullah A. (2023). Pleomorphic Giant Cell Carcinoma of the Prostate: A Case Report and Mini-Review of the Literature. *Medicine International*.

[23] El-Zaatari Z. M., Thomas J. S., Divatia M. K. (2021). Pleomorphic Giant Cell Carcinoma of Prostate: Rare Tumor With Unique Clinicopathological, Immunohistochemical, and Molecular Features. *Annals of Diagnostic Pathology*.

